# Incorporating the soil environment and microbial community into plant competition theory

**DOI:** 10.3389/fmicb.2015.01066

**Published:** 2015-10-08

**Authors:** Po-Ju Ke, Takeshi Miki

**Affiliations:** ^1^Department of Biology, Stanford UniversityStanford, CA, USA; ^2^Institute of Oceanography, National Taiwan UniversityTaipei, Taiwan; ^3^Research Center for Environmental Changes, Academia SinicaTaipei, Taiwan

**Keywords:** coexistence, litter-mediated feedback, Lotka-Volterra model, microbial-mediated feedback, invasibility analysis, spatial-explicit model

## Abstract

Plants affect microbial communities and abiotic properties of nearby soils, which in turn influence plant growth and interspecific interaction, forming a plant-soil feedback (PSF). PSF is a key determinant influencing plant population dynamics, community structure, and ecosystem functions. Despite accumulating evidence for the importance of PSF and development of specific PSF models, different models are not yet fully integrated. Here, we review the theoretical progress in understanding PSF. When first proposed, PSF was integrated with various mathematical frameworks to discuss its influence on plant competition. Recent theoretical models have advanced PSF research at different levels of ecological organizations by considering multiple species, applying spatially explicit simulations to examine how local-scale predictions apply to larger scales, and assessing the effect of PSF on plant temporal dynamics over the course of succession. We then review two foundational models for microbial- and litter-mediated PSF. We present a theoretical framework to illustrate that although the two models are typically presented separately, their behavior can be understood together by invasibility analysis. We conclude with suggestions for future directions in PSF theoretical studies, which include specifically addressing microbial diversity to integrate litter- and microbial-mediated PSF, and apply PSF to general coexistence theory through a trait-based approach.

## Introduction

One of the fundamental goals of plant ecology is to understand the maintenance mechanisms of plant species coexistence (Chesson, 2000). Many hypotheses have been proposed, including the resource competition theory (Hsu et al., 1977; Tilman et al., 1981), two-way trade-off models (Tilman, 1994), and the three-way trade-off strategy model (Grime, 1977). Other theories, such as the neutral theory (Hubbell, 1997) and the metacommunity theory (Leibold et al., 2004), address stochastic and spatial processes as well as deterministic processes. These theoretical frameworks and subsequent accepted viewpoints in plant ecology implicitly rely on environmental determinism (i.e., a unidirectional effect of the abiotic environment on the plant community and unidirectional adaptation of organisms to the given environment). This concept is widely accepted despite the fact Tansley (1935), a plant ecologist, first proposed the term “ecosystem” precisely to address feedback among organisms and the abiotic environment. The resource competition theory (Tilman, 1982) examines the effect of individuals on environmental resource availability, however its competitive outcome depends on rates of external resource supply. An alternative investigative approach in plant community ecology, plant-soil-feedback (PSF), emphasizes the bidirectional interaction between growth limiting factors in soil and plant population and community dynamics (Berendse, 1994; Bever et al., 1997). Plants can affect soil properties, which in turn alter plant growth, survival, reproduction, and competition, subsequently acting as the driving forces for community composition and function. In this sense, Jones et al. (1994) appropriately characterized plants as fundamental ecosystem engineers.

Theoretical advances in PSF research have primarily focused on two major drivers: soil microbes and soil nutrients (see Ehrenfeld et al., 2005 for other factors, and van der Putten et al., 2013 for a comprehensive empirical review). Soil microbes interact with plants, providing diverse functional roles and are responsible for “microbial-mediated” PSF. Plants ‘cultivate’ local microbial communities surrounding plant roots (i.e., species-specific root-associated microbes, including beneficial and detrimental groups), which in turn affects plant performance (Bever et al., 1997; Bever, 2003). On the other hand, the feedback mechanisms mediating soil nutrient availability and the subsequent nutrient cycling between above- and below-ground ecosystem components are often referred to as “litter-mediated” PSF. Soil nutrient availability is determined by organic carbon and plant litter mineralization, which is controlled by plant species specific traits (e.g., litter production rate and its chemistry, Binley and Giardina, 1998) and can influence plant competition outcome (Berendse et al., 1987, 1989; Berendse, 1994). Although, the two mechanisms are commonly discussed individually, under some scenarios, the two main mechanisms are shown to interact and are in fact tightly linked. For example, litter-mediated PSF is also mediated by soil microbial decomposers such as saprophytic bacteria and fungi (Miki et al., 2010; Miki, 2012).

Although, empirical studies have increased our general understanding of PSF, many ecological models are constructed to examine PSF processes for specific species or ecosystems. General PSF mathematical theory remains under-explored, thus hindering comprehensive understanding of the importance of PSF and the roles of belowground microbial-mediated processes in structuring terrestrial ecosystems. Clearly, PSF theory requires development to better understand various plant community properties, including the number of coexisting species (e.g., Molofsky and Bever, 2002; Mazzoleni et al., 2010), species abundance and distribution (e.g., Mack and Bever, 2014), transient successional patterns (e.g., Fukami and Nakajima, 2013), and species spatial and temporal distribution (e.g., Molofsky et al., 2002).

In this article, we summarize how PSF concepts have been integrated into ecological modeling studies, with particular focus on topics related to plant species coexistence and spatio-temporal dynamics (see Table 1 for a list of theoretical studies along with their corresponding modeling framework category). We subsequently reanalyzed two fundamental mechanistic PSF models (i.e., Berendse, 1994; Bever et al., 1997), which are the foundation of current theoretical models, and compared them with the classic Lotka-Volterra competition model. Our results demonstrated how the application of invasibility analysis elucidated behaviors of different PSF models by generating coexistence criteria with similar ecological interpretation. Moreover, invasibility analysis provided superior ideas for soil-centered (or microbe-centered) views in PSF studies, resulting in a revised PSF metric which will aid in future theoretical development in microbial ecology. Finally, we proposed new research directions to integrate litter- and microbial-mediated PSF, and apply trait-based modeling approaches to predict species' PSF and coexistence.

**Table 1:** **Theoretical plant-soil feedback models reviewed in this article**.

**Author (year)**	**Main PSF mechanisms (model type)^†^**	**Study detail (plant community processes)^§^**
Adler and Muller-Landau, 2005	Microbial-PSF^a^	Plant and natural enemy dispersal distance (SS, SD)
Aguilera, 2011	Microbial-PSF^b^	Density-dependency of plant microbe interactions (PI)
Berendse et al., 1987	Litter-PSF^b^	Species-specific litter chemistry (SC)
Berendse et al., 1989	Litter-PSF^b^	Species-specific litter chemistry (SC)
Berendse, 1994	Litter-PSF^b^	Species-specific litter chemistry (SC)
Bever et al., 1997	Microbial-PSF^b^	LV-type plant-microbe interactions (SC)
Bever, 1999	Microbial-PSF^b^	LV-type plant-microbe interactions (SC)
Bever, 2003	Microbial-PSF^b^	LV-type plant-microbe interactions (SC)
Bever et al., 2010	Microbial-PSF^b^	Microbial-mediated plant niche partitioning and PSF (SC)
Bonanomi et al., 2005	No specific mechanism^a^	Negative PSF and population dynamics (SC, SD)
Clark et al., 2005	Litter-PSF^b^	Species-specific litter chemistry (SC)
Daufresne and Hedin, 2005	Litter-PSF^b^	Resource ratio hypothesis and nutrient cycling (SC)
Dickie et al., 2005	Microbial-PSF^a^	Distance-dependent interaction strength (SS)
Eppinga et al., 2006	Microbial-PSF^b^	Nonlinear LV-type plant-microbe interactions (PI)
Eppinga et al., 2011	Litter-PSF^b^	Species-specific litter chemistry and trait evolution (PI)
Eppstein et al., 2006	No specific mechanism^a^	Community dynamics and frequency dependency (SS, SC)
Eppstein and Molofsky, 2007	No specific mechanism^b^	Invasion dynamics and frequency dependency (PI, SC)
Fukami and Nakajima, 2013	No specific mechanism^a^	Transient dynamics and delayed convergence (SD)
Fukano et al., 2013	Microbial-PSF^c^	Disturbance regime (PI)
Kulmatiski et al., 2011	Microbial-PSF^b^	Multi-species LV-type plant microbe interaction (SC)
Kulmatiski et al., 2012	Microbial-PSF^c^	Multi-species biomass-explicit plant-microbe interaction (BEF)
Loeuille and Leibold, 2014	No specific mechanism^a^	Species diversification and macro-ecological patterns (SD)
Levine et al., 2006	Microbial-PSF^d^	Spatial scale and invasion velocity (PI, SS)
Mack and Bever, 2014	Microbial-PSF^a^	Plant dispersal and PSF interactions scale (SRA, SS)
Mangan et al., 2010	Microbial-PSF^a^	Negative PSF and Janzen-Connell hypothesis (SD, SRA)
Mazzoleni et al., 2010	Litter-PSF^b^	Autotoxicity and latitudinal diversity gradient (SD)
Miki and Kondoh, 2002	Litter-PSF^b^	Species-specific litter chemistry (PI, SC)
Miki et al., 2010	Litter- and microbial- PSF^b^	Decomposer diversity (SC)
Miki, 2012	Litter- and microbial- PSF^b^	Decomposer diversity (PI)
Molofsky et al., 2001	No specific mechanism^a^	Coexistence under positive PSF (SS, SC)
Molofsky and Bever, 2002	No specific mechanism^a^	Positive PSF and unsuitable habitats (SS, SD)
Molofsky et al., 2002	No specific mechanism^a^	Plant dispersal and PSF interactions scale (SS, SC)
Mordecai, 2013a	Microbial-PSF^c^	Generalist pathogen and pathogen spillover (SC)
Mordecai, 2013b	Microbial-PSF^c^	Generalist pathogen and pathogen spillover (SC)
Mordecai, 2015	Microbial-PSF^c^	Generalist pathogen and storage effect (SC)
Petermann et al., 2008	Microbial-PSF^a^	Janzen-Connell hypothesis (SD)
Revilla et al., 2013	Microbial-PSF^b^	LV-type plant-microbe interactions (SC)
Schnitzer et al., 2011	No specific mechanism^b^	LV-type plant-microbe interactions (BEF)
Sedio and Ostling, 2013	Microbial-PSF^a^	Natural enemy host specificity and Janzen-Connell hypothesis (SD)
Suding et al., 2013	No specific mechanism^a^	Invasion dynamics and enemy release (PI)
Turnbull et al., 2010	Microbial-PSF^c^	Invasive species spread (PI)
Umbanhowar and McCann, 2005	Microbial-PSF^b^	Plant-mycorrhizal fungi interactions (SC)
Zee and Fukami, 2015	No specific mechanism^a^	Species loss following habitat fragmentation (SD)

*Studies are selected if it considers the effect of microbial-mediated PSF and/or litter-mediated PSF on plant competition outcome or community structure. As a result, models investigating the effect of plant-microbial interaction and nutrient cycling on the growth of a single plant species, as well as those focusing on disease dynamics, are not included*.

† Model type:

a stochastic cellular automata;

b ordinary differential equations;

c difference equations;

d *integrodifference equations*.

§ *Plant community process: SC, species coexistence; PI, plant invasion; BEF, biodiversity-ecosystem functioning relationship; SRA, species relative abundance; SD, species diversity; SS, spatial structure*.

## Theoretical development and modeling achievements for PSF

### PSF effects on plant competition outcome and species coexistence

Theoretical PSF studies were first proposed to examine how PSF contributes to the coexistence of competing plant species. Bever et al. (1997) (hereafter Bever's model) conducted pioneering research leading to the microbial-mediated PSF model, which incorporated reciprocal interactions and frequency dependency among plants and soil microbial communities. Bever's model proposed that the final fate of a plant community can be predicted by the overall effect of soil microbial communities on both plant species (i.e., an interaction coefficient, *I*_*S*_). Bever's model predicted that two plant species can coexist cyclically due to microbial-mediated PSF when both species generate PSF which decreases its relative growth rate (i.e., negative microbial-mediated PSF in terms of negative *I*_*S*_). However, the model predicted that single species dominance is reached when both plants species generate PSF which increases its relative growth rate (i.e., positive microbial-mediated PSF in terms of positive *I*_*S*_). It is important to note that the community-level outcome of PSF (i.e., predicted by the sign of *I*_*S*_) cannot be directly inferred by the interaction type between soil microbes and its host (Bever et al., 1997). For example, if the mycorrhizal fungi delivers more benefit to the competitor compared to that to its host, competing plant species can coexist as PSF would decrease the relative growth rate of its host (i.e., negative *I*_*S*_ despite positive plant-microbe interaction; Bever, 1999, 2002; Umbanhowar and McCann, 2005). Likewise, pathogens can increase the relative growth rate of its host and result in single species dominance if it has stronger suppression on the growth of the competitor (i.e., positive *I*_*S*_ despite negative interaction with its host). A later version of Bever's model incorporated PSF into a two species Lotka-Volterra model and demonstrated coexistence can be promoted by a negative PSF, even under strong competitive interactions and fitness differences between the two plant species (Bever, 2003). Individual-based simulation models incorporating the PSF concept made similar predictions (Bonanomi et al., 2005; Petermann et al., 2008), and further suggested that the magnitude of population oscillations depends on negative PSF strength. However, Revilla et al. (2013) performed a complete analysis of Bever's model and suggested population cycling under negative PSF (i.e., in terms of negative *J*_*S*_, a modified version of *I*_*S*_ in Bever's model) might occur in the form of heteroclinic cycles, which can enable stochastic extinction in real empirical systems. Recent theoretical studies with an emphasis on microbial-mediated PSF have also extended Bever's model to multiple species (Bonanomi et al., 2005; Petermann et al., 2008; Kulmatiski et al., 2011, 2012). For example, a three-species version of Bever's model showed PSF played a critical role in predicting rank order abundance of experimental plant communities, and the PSF model made better predictions compared with a pure competition model (Kulmatiski et al., 2011).

Theoretical studies on litter-mediated PSF investigated the influence of plant litter quality on soil nutrient availability, and how changes in soil nutrient availability alter plant competition (pioneered by Berendse et al., 1987, 1989; Berendse, 1994). When plants compete for a single growth limiting factor in the soil (e.g., inorganic nitrogen), a difference in plant growth response to different nutrient levels (i.e., a tradeoff) is often necessary for litter-mediated PSF to alter competitive outcomes (Miki and Kondoh, 2002; Clark et al., 2005). Berendse (1994) used ordinary differential equations to build simple ecosystem models to demonstrate community-level outcomes depended on a combination of the plant species' litter quality and nutrient uptake strategies. Plant species with growth advantages in nutrient-rich soils reinforced their dominance by producing rapidly decomposing litter. Similarly, plant species more competitive in nutrient-poor sites increased their dominance by producing slowly decomposing litter. Both trait combinations resulted in nutrient availability that favors the resident plant (i.e., positive litter-mediated PSF), leading to competitive exclusion of its competitor (Berendse, 1994), or alternative stable states differing in species composition (Clark et al., 2005) or species richness (Miki and Kondoh, 2002; Miki et al., 2010). In contrast, coexistence was facilitated if plant species influenced the nutrient cycle to reinforce the persistence of its competitor (i.e., negative litter-mediated PSF). Some studies integrated litter-mediated PSF with Tilman's (1982) resource ratio theory to consider multiple limiting factors in the soil and plant stoichiometry (Daufresne and Hedin, 2005; Eppinga et al., 2011). This theoretical framework also demonstrated that whether litter-mediated PSF enhances or suppresses coexistence was dependent on the trait combination of competing plants species (Daufresne and Hedin, 2005). Simple litter-mediated PSF models were also extended to examine more detailed nutrient cycling, emphasizing the importance of environmental factors (Miki and Kondoh, 2002), litter quality attributes other than decomposition rates (e.g., the recycled proportion, Clark et al., 2005), different plant-available nutrient types (Clark et al., 2005; Daufresne and Hedin, 2005), and litter effects other than soil nutrient availability (Eppinga et al., 2011) on community outcomes driven by litter-mediated PSF.

### PSF models that go beyond species coexistence

Recent theoretical studies go beyond discussing coexistence of few plant species, and have applied PSF as a mechanism to explain other macro-scale community patterns (see Bever et al., 2010; van der Putten et al., 2013 and references therein). The relationship between PSF and plant diversity is one topic that has received a great deal of interest. Many empirical studies revealed negative microbial-mediated PSF acted as a mechanism for the Janzen-Connell hypothesis (Janzen, 1970; Connell, 1971), contributing to negative density-dependent (Bell et al., 2006; Yamazaki et al., 2008; Bagchi et al., 2010, 2014) and distance-dependent (Augspurger, 1983; Packer and Clay, 2000; Swamy and Terborgh, 2010) seedling mortality. These mortality patterns resulted from negative PSF, which enhanced plant diversity; and simulation models suggested the greatest diversity was attained when natural enemies were host-specific (Sedio and Ostling, 2013). Furthermore, a species' relative abundance in a community was predicted by the PSF strength it experienced; plant species with lower abundance suffered stronger negative PSF (Klironomos, 2002; Mangan et al., 2010; but see Reinhart, 2012). Mangan et al. (2010) applied a spatially explicit cellular automata model to confirm the positive relationship between species' PSF strength and species relative abundance by parameterizing the model with field-measured PSF strength. Additional work on the model confirmed the positive relationship was robust for different forms of life-history tradeoffs (e.g., tradeoffs between mortality and establishment rates) (Mangan et al., 2010; Mack and Bever, 2014).

Large-scale studies suggested that the stronger negative microbial-mediated PSF at lower latitude regions contributed to its higher plant species richness (Johnson et al., 2012). Mazzoleni et al. (2010) constructed an ecosystem model considering “resource-waste” (i.e., “autotoxicity” via intra-specific toxic compounds, Mazzoleni et al., 2007) during litter decomposition as a source for negative litter-mediated PSF. Autotoxicity has the potential to suppress plant growth directly by harming plant tissue or indirectly by exacerbating detrimental pathogen effects (Mazzoleni et al., 2007, 2010; Bonanomi et al., 2010). The ecosystem model indicated that as litter decomposition rate increased from higher to lower latitude, so did the level of autotoxicity, generating stronger negative density-dependency and supporting higher species richness at lower latitudes. The model also showed that washing of autotoxicity removed negative PSF effects and decreased species richness, a potential mechanism to explain differences in plant species richness between flooded and non-flooded communities at the same latitude (Mazzoleni et al., 2010).

At the interface between community and ecosystem ecology, PSF was also proposed to contribute to the biodiversity-ecosystem functioning relationship, with particular focus on productivity (i.e., higher plant diversity leads to increased productivity, also known as overyielding). Loreau and Hector (2001) attributed an asymptotic productivity increase to either sampling effect or niche complementary due to a rise in species diversity (Loreau and Hector, 2001; but see Hector et al., 1999; Miki, 2009; Cardinale et al., 2012 for diversity-productivity patterns other than asymptotically increase). Soil microbes, particularly host-specific pathogens, were recently proposed as another potential mechanism involved in the diversity-productivity relationship. Studies showed that negative density-dependent effect of pathogens were diluted as plant species diversity increased since the proportion of self-cultivated soil decreased, resulting in higher plant productivity (Maron et al., 2011; Schnitzer et al., 2011). Empirical studies supported this mechanism; compared with monocultures, plants overyield when grown in a soil mixture cultivated with different plant species (Kulmatiski et al., 2012; Hendriks et al., 2013), while sterilizing the soil eliminated the positive relationship (Maron et al., 2011; Schnitzer et al., 2011). In addition, Hendriks et al. (2015) found spatial heterogeneity in soil biota created by highly diverse communities also contributed to plant avoidance of host-specific pathogens. Schnitzer et al. (2011) used a simple Lotka-Volterra type model to illustrate that the classic asymptotic diversity-productivity relationship appeared only in the presence of host-specific pathogens. The pathogen effects were stronger, and the plant productivity saturation point occurred at higher plant diversity, when operating together with niche complementary (i.e., comparing neutral and non-neutral models). Based on Bever's PSF model, Kulmatiski et al. (2012) developed a biomass-explicit multi-species PSF model to directly examine the influence of PSF on biomass production. Their model suggested that dilution of species-specific soil biota effects, resulting from increased plant diversity, can result in over- and under-yielding, depending on the sign of species' PSF (i.e., negative or positive, respectively). In addition, the negative relationship between PSF strength and over-yielding became stronger with increasing species richness. Empirical results supported model predictions, however results also indicated further information regarding plant community structure (e.g., presence or absence of nitrogen-fixing plants) would provide useful information for future studies (Kulmatiski et al., 2012). Although, theoretical studies linking microbial-mediated PSF and diversity-productivity relationships are growing, to our knowledge models related to this topic have not explicitly considered litter-mediated PSF and nutrient cycling.

Finally, the impact of PSF on invasion success has been another rapidly growing research area (empirical studies reviewed in Mitchell et al., 2006; Reinhart and Callaway, 2006; Inderjit and van der Putten, 2010; Suding et al., 2013). In general, PSF facilitates invasion when exotic plant species experience weaker negative PSF (or benefit from stronger positive PSF) compared to native plant species (Reinhart and Callaway, 2006). Keane and Crawley (2002) proposed the “enemy-release hypothesis,” which stated that by migrating from their native range, exotic species escaped species-specific specialized natural pathogens. Consequently, exotic species experienced reduced negative microbial-mediated PSF in the new region and became successful invaders (Keane and Crawley, 2002; Klironomos, 2002; Mitchell and Power, 2003; Callaway et al., 2004). Further, theoretical studies suggested the effectiveness of enemy-release depended on the diversity of the native community (Turnbull et al., 2010), the functional response between plant growth and soil microbial density (Aguilera, 2011), the disturbance regime (Fukano et al., 2013), and the invader competitive ability on the native species' cultivated soil environment (Eppstein and Molofsky, 2007; Turnbull et al., 2010; Suding et al., 2013). In some cases, soil-borne pathogens might still facilitate invasion, even if the invader is not enemy-released. This happens when the invader attracts generalist pathogens, which have stronger negative effects on native plant species, a scenario termed the “enemy-accumulation hypothesis.” This scenario was first proposed by Eppinga et al. (2006) using a non-linear extension of Bever's model to explain the success of *Ammophila arenaria* invasion in California, which was a successful invader despite suffering similar pathogen suppression as that in its native European range (Bever et al., 1997; Beckstead and Parker, 2003). The effects of generalist pathogens on plant invasion have also been studied under the “pathogen spillover” hypothesis, where invasive plant species with greater pathogen tolerance gain advantage by transmitting the shared pathogens to less tolerant native plant species (Beckstead et al., 2010). Pathogen spillover can lead to competitive exclusion of natives, coexistence, or priority effects (Mordecai, 2013a,b). In addition to microbial-mediated PSF, litter-mediated PSF can also influence the outcome of exotic plant invasion. Specific combinations of litter decomposability and nutrient uptake strategies will generate positive litter-mediated PSF for the invader (Miki and Kondoh, 2002). When linking litter-mediated PSF with invasion, studies have also discussed its effect on resources other than soil nutrient. The effect of invader's litter on the local light environment has been of particular interest. Results of studies indicated that invaders' litter accumulation will decrease light availability by shading, while simultaneously increasing soil nutrient availability via litter decomposition. Studies suggested that the combined impact of invader litter facilitated invasion non-additively when the invader is a weaker competitor for soil nutrients but a better competitor for light (Farrer and Goldberg, 2009; Eppinga et al., 2011).

### The importance of spatial scale on PSF effects

One simplification made in many PSF models is the assumption of a well-mixed soil environment, which neglects the fact that plant dispersal and PSF usually operate locally. However, empirical evidence often revealed that the interaction between plants and soil microbes are highly distance-dependent and can influence plant spatial patterning (Augspurger, 1983; Packer and Clay, 2000; Reinhart et al., 2003; Dickie et al., 2005; Swamy and Terborgh, 2010). Eppstein et al. (2006) applied spatially explicit models to investigate the importance of space on PSF outcomes; results differed from the well-mixed models and provided new insights into the value of PSF and plant spatial patterning. In addition, the prediction from Bever's model regarding PSF sign and plant competition outcome had been extensively studied (Bever et al., 1997; Molofsky et al., 2001, 2002; Molofsky and Bever, 2002; also reviewed in Bever, 2003 and Bever et al., 2012). Molofsky et al. (2002) showed that under a spatially explicit modeling framework the prediction that negative PSF facilitated plant coexistence was generally not altered (but see Bever et al., 1997 for the case when two plants exhibited different dispersal ability). Plant coexistence was maintained by negative PSF, while a large array of different spatial patterns was observed, ranging from near random, clumped, to band formation distributions. The spatial patterning of competing plant species depends on the relative spatial scale of dispersal and PSF, as well as the negative PSF strength (Molofsky et al., 2002). However, the prediction that positive PSF leads to single species dominance was altered when considering the spatial aspects of PSF. Studies demonstrated that under certain initial conditions, positive PSF can lead to the formation of self-maintained monomorphic patches, promoting long-term coexistence at the regional scale via the maintenance of spatial heterogeneity (Molofsky et al., 2001; Molofsky and Bever, 2002). Moreover, the probability of species coexistence was greater when positive PSF existed compared with the absence of PSF (i.e., a neutral case), and coexistence was more effectively maintained when positive PSF operated at a local scale (Molofsky et al., 2001; Molofsky and Bever, 2002). This unique prediction was maintained even when the model was generalized to consider asymmetric frequency-dependent strength between competing plant species (Eppstein et al., 2006). Dickie et al. (2005) demonstrated that mycorrhizal-generated positive PSF and plant-plant competition showed different distance-dependent patterns. By integrating empirical evidence into a spatially explicit model, results indicated a net mycorrhizal effect facilitating seedling growth occurred only at low plant densities, thus potentially promoting species coexistence and diversity (Dickie et al., 2005). These theoretical achievements suggested that when spatial structure of interactions was considered, the predicted species monoculture associated with positive PSF was mitigated and coexistence was maintained at larger spatial scales.

In addition to the coexistence patterns between two plant species, other macro-scale community patterns generated by PSF have also been examined under a spatial framework. By considering spatial structure and heterogeneous landscape (i.e., with unsuitable habitats conditions), Molofsky and Bever (2002) suggested that positive PSF can maintain community species diversity for a much longer time period if PSF operated at a local scale. For negative microbial-mediated PSF to effectively promote species richness, local microbial and plant dispersion was required to generate clumped adult distribution and strong negative density-dependency for seedling survival (Adler and Muller-Landau, 2005; Petermann et al., 2008). The positive relationship between species' PSF strength and its relative abundance (Mack and Bever, 2014), as well as evolutionary diversification (Loeuille and Leibold, 2014), were also only observed when the scale of negative PSF and plant dispersal were local. For exotic plant invasion, positive PSF created by the invader can promote its invasiveness when operating at a local scale, despite the counteracting positive PSF created by natives (Eppstein et al., 2006). However, Levine et al. (2006) showed for positive PSF to accelerate invasion velocity, the spatial scale of soil modification must be larger than that of the plant-plant competition.

Former studies typically considered positive or negative PSF separately, however recent models have begun to consider the spatial consequences of a complex PSF scenario (Fukami and Nakajima, 2013), where PSF affects plant growth positively for some species-pair, and negatively for others. Fukami and Nakajima (2013) evaluated compositional variation between patches with different initial compositions, and suggested a complex PSF scenario could maintain the initial variability in species composition for a longer period of time, contributing to regional plant diversity compared to a simpler PSF scenario. A complex PSF scenario also alleviated diversity loss caused by habitat fragmentation, and such buffering effects were greatest when plants dispersed locally. The mechanisms for such a buffering effect is because complex PSF decreased both spatial clustering of species distribution prior to habitat fragmentation and extinction probability after fragmentation, which would both be pronounced in the absence of PSF if plants dispersed locally (Zee and Fukami, 2015). In conclusion, spatially explicit PSF models generated many useful predictions that cannot be revealed if a well-mixed soil environment was assumed, and suggested that the structuring ability of PSF strongly depends on the spatial scale of PSF and plant dispersal.

### Temporal dynamics of PSF and its effect on plant community succession

The influence of PSF has primarily been examined in studies that focused on community properties in a single temporal snapshot, or implicitly assumed the community is at equilibrium (but see Mordecai, 2015, for the effects of generalist pathogens on plant species coexistence through a temporal storage effect). However, PSF also contributes to temporal dynamics and the development of plant communities. Most of our current knowledge in this area derives from successional studies (reviewed in Kardol et al., 2013; van der Putten et al., 2013). The following two concepts were proposed for the roles of PSF during succession: (i) the predictable and directional changes in PSF sign and strength (Kardol et al., 2006); and (ii) random-emergence of PSF (Fukami and Nakajima, 2013). The first dominant concept suggested that during succession, negative PSF was experienced by early-successional plant species and facilitated replacement by late-successional plant species (van der Putten et al., 1993; Berendse, 1994; Bonanomi et al., 2005; Clark et al., 2005). In a well-known empirical example, Kardol et al. (2006) demonstrated that during secondary succession of abandoned grasslands, directional changes in PSF strength occurred and early-successional plant species mainly experienced negative PSF, while late-successional species primarily experienced positive PSF, which stabilized the plant community composition. This directional sequence of PSF occurrence resulted from nonrandom linkage between plants' above- and below-ground PSF-related traits; early-arriving plant species generally exhibited fast growth rates, but poor defense against pathogens, while late-arriving species were typically slow growing and mycorrhizal-associated species (Kardol et al., 2006, 2007, 2013).

While directional change in PSF strength across plant succession (i.e., from negative to positive PSF) has attracted much attention, empirical evidence supporting other hypotheses has also been reported (e.g., Reynolds et al., 2003; Sikes et al., 2012). A new idea proposed by Fukami and Nakajima (2013) focused on the priority effects generated by PSF, and how different PSF scenarios would influence community trajectories among patches with different immigration histories. Their main conclusion was when PSF operated in a complex manner such that the direction and strength of species' PSF was not determined by its successional stage (e.g., PSF for early-successional species were not necessarily negative), initial species variation among patches would be maintained, and more plant species could colonize a patch and persist longer before local extinction. This results in a long-lasting transient phase characterized by high species turnover (i.e., a large number of local colonization and extinction events) and high regional diversity before reaching the final community structure (Fukami and Nakajima, 2013). Therefore, the model indicated that when PSF occurred idiosyncratically, with a weak correlation between its strength and the species' successional stage, PSF delayed succession and the speed for local communities to converge to a late successional stage community. The different successional trajectories resulting from different PSF scenarios represented alternative transient states in plant community development, which is defined as communities varying in structure or function before reaching a stable state (Fukami and Nakajima, 2011). A promising direction for future research is to integrate the directional and random-emergence paradigm for changes in PSF during succession, and test predictions for successional trajectories using chronosequences from different ecosystems.

## Linkage between mechanistic-PSF models and the Lotka-Volterra competition model

The influence of PSF on plant community dynamics has been incorporated into theoretical ecological modeling, however many of these models are phenomenological and not restricted to specific PSF mechanisms (Table 1). In terms of specific PSF mechanisms, it can be traced back to two fundamental models: microbial- (Bever et al., 1997) and litter-mediated PSF (Berendse, 1994). Here, we reintroduce these two mechanistic-PSF models with more unified formulations. We reanalyzed both models using invasibility analysis (Bever, 2003; Revilla et al., 2013) to show how the final community state can be classified into four possible outcomes, a scenario comparable to the classic Lotka-Volterra competition model. We believe this method provides a systematic way to evaluate PSF models, regardless of the specific topics which they originally focused on.

### Lotka-Volterra competition model and invasibility analysis for PSF models

Our analytical framework can first be illustrated by considering the phenomenological Lotka-Volterra competition model for two plant species (with species density represented by *P*_*A*_ and *P*_*B*_, respectively), which is written as follows:

(1) $$\begin{array}{rcl} \frac{dP_{A}}{dt} & = & r_{A}P_{A}\left(1-\frac{P_{A}+c_{B}P_{B}}{T_{P}}\right) \end{array}$$

(2) $$\begin{array}{rcl} \frac{dP_{B}}{dt} & = & r_{B}P_{B}\left(1-\frac{c_{A}P_{A}+P_{B}}{T_{P}}\right). \end{array}$$

Here, *r*_*i*_ represents intrinsic population growth rate for plant species *i* (*i* = *A* or *B*), *T*_*P*_ denotes the carrying capacity for plants (for simplicity, we assumed identical parameter values for both species). Coefficient *c*_*j*_ measures the negative effects of space competition from heterospecies *j* relative to the negative density dependent effect of conspecies *i* (i.e., *j* = *B* or *A* when *i* = *A* or *B*, respectively). If *c*_*j*_ = 1, species are identical in terms of competition for space (i.e., a neutral case when intra- and inter-specific competition have similar magnitudes). The final outcome in Lotka-Volterra type competition can be predicted by applying invasibility analysis. That is, evaluating species' per capita growth rate (i.e., invader fitness) when its density is low while the competitor is at its monoculture equilibrium (Chesson, 2000). When invader fitness is positive, the invasibility criteria is fulfilled and population size increases (or recovers) from low density. A plant species monoculture occurs when only one species can invade the other. Coexistence is achieved when two plant species are mutually invasive (i.e., each species can invade the habitat dominated by the other species and no species can competitively exclude the other), while founder control (i.e., competition outcome depends on the initial plant density conditions) occurs when both invader species are unable to invade the other. From Equations (1) and (2), the invasibility criterion for *P*_*A*_ to invade a monoculture of *P*_*B*_ (i.e., ***E***_*B*_(*P*${}_{A}^{*}$ = 0, *P*${}_{B}^{*}$ = *T*_*P*_)) is *c*_*B*_ < 1, while that for *P*_*B*_ to invade monoculture of *P*_*A*_(i.e., ***E***_*A*_(*P*${}_{A}^{*}$ = *T*_*P*_, *P*${}_{B}^{*}$ = 0)) is *c*_*A*_ < 1. Here, ***E***_*i*_ is the mathematical notation for the plant monoculture equilibrium of *P*_*i*_, and *P*${}_{i}^{*}$ within the parenthesis represents the density of *P*_*i*_ at the specific equilibrium. Therefore, the parameter space of *c*_*A*_ and *c*_*B*_ can be partitioned into the following four regions: (i) *P*_*A*_ monocultures (*c*_*B*_ < 1 but *c*_*A*_ > 1); (ii) *P*_*B*_ monocultures (*c*_*B*_ > 1 but *c*_*A*_ < 1); (iii) coexistence (both *c*_*B*_ and *c*_*A*_ < 1); (iv) founder control (i.e., alternative stable state of either *P*_*A*_ or *P*_*B*_ monoculture; both *c*_*B*_ and *c*_*A*_ > 1). These results are consistent with those obtained from local stability analysis; coexistence was possible when impacts on heterospecific growth are weaker than those on conspecific growth (i.e. *c*_*B*_ and *c*_*A*_ < 1; Chesson, 2000).

The framework of invasibility analysis can be applied to mechanistic-PSF models. Consider a two plant species (i.e., *P*_*A*_ and *P*_*B*_) and two corresponding soil components (e.g., either soil nutrients or microbial communities) that influence plant population dynamics. When the community is at equilibrium for the *P*_*A*_ monoculture (i.e. ***E***_*A*_(*P*${}_{A}^{*}$ > 0, *P*${}_{B}^{*}$ = 0)), the corresponding soil component is determined by the parameters related to the dominant resident species (in this case *P*_*A*_), but is independent of the invader (*P*_*B*_) parameters. The invasion criterion for *P*_*B*_ to invade the *P*_*A*_ monoculture can be derived from its invader fitness. Importantly, this invasibility criterion includes parameters determining how *P*_*B*_ is affected directly by *P*_*A*_ and indirectly by *P*_*A*_-cultivated soil components, but does not include parameters related to how *P*_*B*_ affects the soil. The criterion implies that the important invasion determinants are interactions between resident species and resident-cultivated soil. Therefore, by combining mutual invasibility conditions for PSF models, we can categorize the trait parameter space related to *P*_*A*_- and *P*_*B*_-soil interactions into different consequences of monoculture and/or coexistence.

### Microbial-mediated PSF models and invasibility analyses

**Figure 1:**
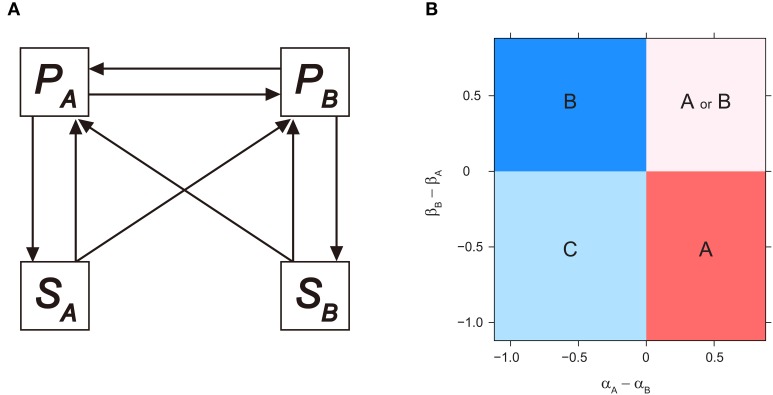
**Invasibility analysis for microbial-mediated PSF model**. **(A)** Model diagram for microbial-mediated PSF model, derived from Bever (2003), see text for model equations. **(B)** Consequences of microbial-mediated PSF on plant competition outcome, as a function of microbial effects on conspecific growth (i.e., α_*A*_ and β_*B*_) minus the effects on heterospecific growth (i.e., α_*B*_ and β_*A*_). *P*_*A*_ or *P*_*B*_ monocultures (i.e., ***E***_***A***_ or ***E***_***B***_) depicted in regions **A** or **B**, respectively. Coexistence of both plant species depicted in region **C**, and alternative stable states of *P*_*A*_ or *P*_*B*_ monocultures depicted in regions **A** or **B**, depending on initial plant density. Numerical simulations were performed by setting α_*B*_ and β_*A*_ as 0.1, and vary α_*A*_ and β_*B*_ sequentially. Other parameters are as follows: *T*_*P*_ = 2.0; *r*_*A*_ = *r*_*B*_ = 2.0; *c*_*A*_ = *c*_*B*_ = 1.0; *v* = 1.0.

Microbial-mediated PSF was first incorporated into the Lotka-Volterra model by Bever et al. (1997) and Bever (2003), and further extended by other studies (Eppinga et al., 2006; Bever et al., 2010; Aguilera, 2011; Kulmatiski et al., 2011; Revilla et al., 2013). Bever's groundbreaking models (Bever et al., 1997; Bever, 2003) captured the bidirectional interaction of PSF; different plant species interacted with the soil microbial community differently and developed a species-specific microbial community association, and changes in microbial communities had differential influences on different plant species (Bever et al., 1997; see Figure 1A for model diagram adapted from Bever, 2003). The microbial community density specifically associated with *P*_*A*_ and *P*_*B*_ was represented by *N*_α_ and *N*_β_, respectively. The dimensionality of the soil microbial community was reduced by focusing on the relative abundance of different soil microbial communities, which were represented by *S*_*A*_ = *N*_α_ ∕ (*N*_α_ + *N*_β_) and *S*_*B*_ = *N*_β_/(*N*_α_ + *N*_β_) for *P*_*A*_- and *P*_*B*_-associated soil microbial communities, respectively (Bever et al., 1997). The model also assumed linear frequency dependency between plants and soil microbial communities (but see Eppinga et al., 2006 and Aguilera, 2011 for a non-linear case). When integrating microbial-mediated PSF in the Lotka-Volterra model, the population dynamics for plants can be written as follows (Bever, 2003):

(3) $$\begin{array}{rcl} \frac{dP_{A}}{dt} & = & r_{A}P_{A}\left(1+\alpha_{A}S_{A}+\beta_{A}S_{B}-\frac{P_{A}+c_{B}P_{B}}{T_{P}}\right) \end{array}$$

(4) $$\begin{array}{rcl} \frac{dP_{B}}{dt} & = & r_{B}P_{B}\left(1+\alpha_{B}S_{A}+\beta_{B}S_{B}-\frac{c_{A}P_{A}+P_{B}}{T_{P}}\right). \end{array}$$

The parameters α_*A*_ and α_*B*_ measure how *S*_*A*_ influences the per-capita growth rate of *P*_*A*_ and *P*_*B*_, respectively, while β_*A*_ and β_*B*_ measure how *S*_*B*_ influences the per-capita growth rates of *P*_*A*_ and *P*_*B*_, respectively. The feedback parameters α_*A*_ and β_*B*_ denote the effect of microbial community on its associated host and are termed direct (or intraspecific) PSF. Alternatively, the microbial community can affect its associated host by influencing the growth of competing plant species. Such interactions are termed indirect (or interspecific) PSF, and are indicated by α_*B*_ and β_*A*_. The sign and magnitude of these feedback parameters are determined by both plant and microbial traits that influence microbial composition (e.g., detrimental pathogens or beneficial mycorrhiza), and interaction strength between plants and microbes (e.g., host specificity of microbes) (Bever et al., 1997, 2010; Bever, 2002).

Under the assumption of linear frequency dependency, the sizes of soil microbial communities (i.e., *N*_α_ and *N*_β_) were assumed to increase linearly with the relative abundance of its associated host plant as:

(5) $$\begin{array}{rcl} \frac{dN_{\alpha }}{dt} & = & \frac{P_{A}}{P_{A}+P_{B}}\cdot N_{\alpha } \end{array}$$

(6) $$\begin{array}{rcl} \frac{dN_{\beta }}{dt} & = & \nu \frac{P_{B}}{P_{A}+P_{B}}\cdot N_{\beta }, \end{array}$$

where *v* represents the relative influence of *P*_*B*_ on the microbial community against that of *P*_*A*_. One way to interpret *v* is the rate that *P*_*B*_, relative to *P*_*A*_, converts the microbial community to its specific associated composition (Kulmatiski et al., 2011), which can be determined by plant exudate and microbial response (reviewed in Bais et al., 2006; Bever et al., 2012). Since, *S*_*A*_ + *S*_*B*_ = 1, the dynamics of relative abundance in different microbial communities can be derived by applying the quotient rule of calculus to Equation (5) as follows:

(7) $$\begin{array}{l} \frac{dS_{A}}{dt}=S_{A}\left(1-S_{A}\right)\left(\frac{P_{A}}{P_{A}+P_{B}}-v\frac{P_{B}}{P_{A}+P_{B}}\right). \end{array}$$

In regard to plant species composition, monoculture equilibrium of either *P*_*A*_ or *P*_*B*_ are derived from Equations (3), (4), and (7) as ***E***_*A*_(*P*${}_{A}^{*}$ = *T*_*P*_ (1 + α_*A*_), *P*${}_{B}^{*}$ = 0) and ***E***_*B*_(*P*${}_{A}^{*}$ = 0, *P*${}_{B}^{*}$ = *T*_*P*_ (1 + β_*B*_)), respectively. The corresponding soil equilibrium states are *S*${}_{A}^{*}$ = 1 and *S*${}_{A}^{*}$ = 0, respectively. Based on local stability analysis, Bever et al. (1997) proposed that the community outcome was predicted by the sign of an interaction coefficient, *I*_*S*_, which was defined as *I*_*S*_ = α_*A*_ − β_*A*_ − α_*B*_ + β_*B*_. The necessary condition for coexistence to be maintained by PSF was *I*_*S*_ < 0. In contrast, PSF resulted in a monoculture of either plant species when *I*_*S*_ > 0. Here, applying a similar invasibility analysis used in Bever (2003) and Revilla et al. (2013), we calculated plant *P*_*A*_ would invade the *P*_*B*_ monoculture (i.e., ***E***_***B***_) when:

(8) $${\underset{P_{A}\to +0}{\lim}\frac{1}{P_{A}}\frac{dP_{A}}{dt}|}_{E_{B}}=r_{A}\left[1+\beta_{A}-\frac{c_{B}T_{P}\left(1+\beta_{B}\right)}{T_{P}}\right]>0.$$

As we were interested in the effects of PSF strength on the invasion criteria, we assume *c*_*A*_ = *c*_*B*_ = 1 (i.e., a neutral case such that the two plants are identical in terms of competition for space). The invasibility criterion for *P*_*A*_ can be written as β_*B*_ − β_*A*_ < 0. This criterion implies that *P*_*A*_ can invade ***E***_*B*_ if the dominating *P*_*B*_-cultivated soil has less facilitative (or more negative) effects on *P*_*B*_ compared to that on the invading *P*_*A*_ (i.e., *P*_*B*_-cultivated soil has a relatively negative effect on *P*_*B*_ compared to that on *P*_*A*_). On the other hand, plant *P*_*B*_ can invade the monoculture of *P*_*A*_ (i.e., ***E***_***A***_) when:

(9) $${\underset{P_{B}\to +0}{\lim}\frac{1}{P_{B}}\frac{dP_{B}}{dt}|}_{E_{A}}=r_{B}\left[1+\alpha_{B}-\frac{c_{A}T_{P}\left(1+\alpha_{A}\right)}{T_{P}}\right]>0,$$

which gives the invasion criterion as α_*A*_ – α_*B*_ < 0 when *c*_*A*_ = *c*_*B*_ = 1. Consequently, *P*_*B*_ can invade ***E***_*A*_ if the dominant *P*_*A*_-cultivated soil has a relatively negative effect on *P*_*A*_. Note that these criteria are determined by the sign of direct PSF (i.e., α_*A*_ and β_*B*_) minus indirect PSF (i.e., α_*B*_ and β_*A*_), and can be interpreted as the differential effect of the cultivated soil on its host compared to the effects on the other plant species. The parameter space of α_*A*_ – α_*B*_ and β_*B*_ – β_*A*_ can therefore be partitioned into four regions based on these invasibility criteria (also confirmed by numerical simulations, Figure 1B). Coexistence (region C in Figure 1B) is possible when both plant species support a microbial community which has stronger facilitative effects on the competitor compared to that on itself, or stronger suppressive effects on itself than on the competitor (i.e., both α_*A*_ – α_*B*_ and β_*B*_ – β_*A*_ < 0). Alternatively, microbial-mediated PSF leads to a plant species monoculture (region A and B in Figure 1B) when only one plant cultivates the microbial community to have a positive effect on itself (i.e., only β_*B*_ – β_*A*_ or α_*A*_ – α_*B*_ < 0 for *P*_*A*_- and *P*_*B*_-monoculture, respectively). Finally, when both plant species experience positive feedback from its associated microbial community (i.e., both α_*A*_ – α_*B*_ and β_*B*_ – β_*A*_ > 0), the system generates alternative stable state of monoculture of either plant species (region A or B in Figure 1B). Note that by relaxing the *c*_*A*_ = *c*_*B*_ = 1 assumption, similar simple boundaries are produced, although the invasion criteria becomes β_*B*_ – β_*A*_ < 1 - *c*_*B*_ for *P*_*A*_ and α_*A*_ – α_*B*_ < 1- *c*_*A*_ for *P*_*B*_ (see also Bever, 2003 and Revilla et al., 2013 for model analysis when relaxing this assumption).

We argue that applying the invasibility analysis to Bever's fundamental microbial-mediated PSF model (Bever, 2003) provides a more transparent interpretation of PSF. For example, consider conditions where α_*A*_ > α_*B*_ and β_*A*_ > β_*B*_ (i.e., *P*_*A*_ has greater growth advantages compared to *P*_*B*_, despite competitive equivalence for space). Dominance of *P*_*A*_ can be successfully predicted by criteria derived from invasibility analysis, but not by the sign of *I*_*S*_ (e.g., positive coexistence equilibrium does not exist under some parameter region despite *I*_*S*_ < 0). In addition, unlike local stability analysis, which can only pinpoint the local asymptotically stable criteria of the coexistence equilibrium, invasibility analysis relies on species' per capita growth rate when rare (i.e., at low population density) and identifies the criteria for either stable or fluctuating coexistence (Chesson, 2000).

### Litter-mediated PSF models and invasibility analysis

**Figure 2:**
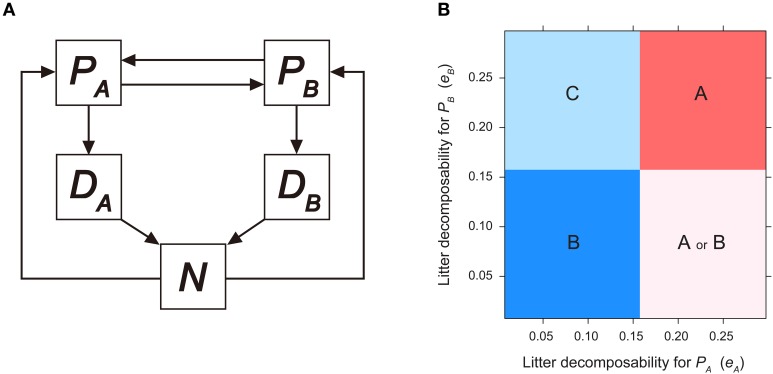
**Invasibility analysis for litter-mediated PSF model**. **(A)** Model diagram for litter-mediated PSF model derived from Berendse (1994), see text for model equations. **(B)** Consequences of litter-mediated PSF on plant competition outcome, as a function of plant litter decomposability (i.e., *e*_*A*_ and *e*_*B*_). Parameters are as follows: *T*_*P*_ = 10.0; *T*_*N*_ = 20.0; *v*_*A*_ = 2.0; *v*_*B*_ = 1.0; *K*_*A*_ = 2.0; *K*_*B*_ = 0.3; *m*_*A*_ = *m*_*B*_ = *m*=0.3; *b*_*A*_ = *b*_*B*_ = *b* = 1.0; *c*_*A*_ = *c*_*B*_ = 1.0. See Figure 1 legend for region definition.

In addition to microbial-mediated PSF, another branch of mechanistic-PSF models focus on plants ability to alter soil biochemical properties through litter-mediated PSF (Binley and Giardina, 1998). Berendse (1994, see also Berendse et al., 1987, 1989) proposed the litter-mediated PSF model, which was further extended by other studies (Miki and Kondoh, 2002; Clark et al., 2005; Miki et al., 2010; Eppinga et al., 2011). A simplified version of the model (Figure 2A, modified from Berendse, 1994) incorporates plant growth limitations by soil nutrients and nutrient cycling processes into the Lotka-Volterra model as follows:

(10) $$\begin{array}{rcl} \frac{dP_{A}}{dt} & = & \frac{v_{A}N}{K_{A}+N}\left(1-\frac{P_{A}+c_{B}P_{B}}{T_{P}}\right)P_{A}-m_{A}P_{A} \end{array}$$

(11) $$\begin{array}{rcl} \frac{dP_{B}}{dt} & = & \frac{v_{B}N}{K_{B}+N}\left(1-\frac{c_{A}P_{A}+P_{B}}{T_{P}}\right)P_{B}-m_{B}P_{B} \end{array}$$

Here, *v*_*i*_ and *K*_*i*_(*i* = *A* or *B*) represent the maximum rate and half-saturation constant for plant species *i* (*i* = *A* or *B*) to uptake plant-available nutrients in the soil, *N*. The term *v*_*i*_*N*/(*K*_*i*_ + *N*) in Equations (10) and (11) represent how the per capita growth rate of *P*_*i*_ is influenced by nutrient availability; the term in the parenthesis denotes growth limitation due to space competition, and the last term represents plant mortality (with species-specific mortality rate, *m*_*i*_). To ensure potential coexistence of plant species, in the following analysis we considered trade-offs between nutrient uptake strategies: *P*_*A*_ (or *P*_*B*_) tends to be competitively superior with high nutrient (low nutrient) levels (Miki and Kondoh, 2002; Clark et al., 2005). When parameterizing the model, we set *v*_*A*_ > *v*_*B*_ while *v*_*A*_/*K*_*A*_ < *v*_*B*_/*K*_*B*_.

Dead plant materials from *P*_*A*_ and *P*_*B*_ enter the plant-unavailable litter pool, with nutrient content denoted by *D*_*A*_ and *D*_*B*_, respectively. The amount of nutrient transferred was calculated by the number of dead individuals multiplied by nutrient content per individual, *b*_*i*_ (i.e., the first term in Equations (12) and (13)).

(12) $$\begin{array}{rcl} \frac{dD_{A}}{dt} & = & b_{A}m_{A}P_{A}-e_{A}D_{A} \end{array}$$

(13) $$\begin{array}{rcl} \frac{dD_{B}}{dt} & = & b_{B}m_{B}P_{B}-e_{B}D_{B} \end{array}$$

(14) $$\begin{array}{l} \frac{dN}{dt}=e_{A}D_{A}+e_{B}D_{B}-\frac{b_{A}v_{A}N}{K_{A}+N}\left(1-\frac{P_{A}+c_{B}P_{B}}{T_{P}}\right)P_{A}\\ \;-\frac{b_{B}v_{B}N}{K_{B}+N}\left(1-\frac{c_{A}P_{A}+P_{B}}{T_{P}}\right)P_{B}. \end{array}$$

The second term in Equations (12) and (13) represents nutrient release from biological components through plant litter decomposition, with species-specific decomposition rate, *e*_*i*_. For simplicity, it is assumed that litter chemical quality determines its decomposition rate (Cornwell et al., 2008), excluding the role of saprophytic microbes (but see Knops et al., 2002; Miki et al., 2010; Miki, 2012). Mineralized nutrients are returned to the plant-available nutrient pool and taken up by plants for population growth. Under this simple framework, the dynamic system was assumed to be closed, such that *b*_*A*_*P*_*A*_(*t*) + *b*_*B*_*P*_*B*_(*t*) + *D*_*A*_(*t*) + *D*_*B*_(*t*) + *N*(*t*) = *const*. ≡ *T*_*N*_, where *T*_*N*_ represents the system's total nutrient content (but see Menge et al., 2009 for an opened ecosystem model).

The community outcome of the litter-mediated PSF model can be characterized by combining local stability analysis and invasibility analysis. We set *c*_*A*_ = *c*_*B*_ = 1, *m*_*A*_ = *m*_*B*_ = *m*, and *b*_*A*_ = *b*_*B*_ = *b* to examine the effects of litter decomposition rates, *e*_*A*_ and *e*_*B*_. The monoculture equilibrium of plant species *i* exhibits the following form: ***E***_*i*_(*P*${}_{i}^{*}$ = *T*_*P*_ − *T*_*P*_*m*(*K*_*i*_ + *N*${}_{i}^{*}$)/(*v*_*i*_ *N*${}_{i}^{*}$), *P*${}_{j}^{*}=$ 0), where *N*${}_{i}^{*}$ is the corresponding equilibrium for soil nutrient pool when the system is at monoculture of *P*_*i*_ (*i* = *A* or *B*). By applying invasibility analysis to the litter-mediated PSF model, we derive that plant *P*_*B*_ can invade the *P*_*A*_-monoculture (i.e., ***E***_*A*_) when:

(15) $$\begin{array}{l} N_{A}^{*}<\frac{K_{A}v_{A}-K_{B}v_{B}}{v_{A}-v_{B}}\equiv \hat{N}, \end{array}$$

while the condition for *P*_*A*_ to invade ***E***_*B*_ is calculated as follows:

(16) $$\begin{array}{l} N_{B}^{*}>\hat{N}. \end{array}$$

To solve *N*${}_{i}^{*}$ (and corresponding plant litter decomposition rate), which fulfill Equations (15) and (16), we substitute Equations (10–13) into Equation (14) to derive the quadratic equation of *N*${}_{i}^{*}$ for each monoculture equilibrium as follows:

(17) $$\begin{array}{c} \begin{array}{l} f_{i}\left(N_{i}^{*}\right)\equiv \left(e_{i}v_{i}\right)N_{i}^{*2}+\left[\left(e_{i}+m\right)\left(v_{i}-m\right)bT_{P}-e_{i}v_{i}T_{N}\right]N_{i}^{*}\\ \;+\left[-K_{i}bmT_{P}\left(e_{i}+m\right)\right]=0. \end{array} \end{array}$$

The above equation has one unique positive root due to the positive quadratic coefficient but negative constant term. The inequality *f*_*A*_($\hat{N}$) > 0 must be satisfied to obtain the *N*${}_{A}^{*}$ fulfilling the invasibility criterion for *P*_*B*_ (i.e., Equation (15)), resulting in the following:

(18) $$\begin{array}{rcl} e_{A} & < & \frac{mbT_{P}v_{A}\left[\left(K_{A}v_{B}-K_{B}v_{A}\right)-m\left(K_{A}-K_{B}\right)\right]}{v_{A}\left(K_{A}v_{B}-K_{B}v_{A}\right)\left\{T_{N}-\left[\left(v_{A}-m\right)-\frac{\left(v_{A}-v_{B}\right)K_{A}m}{\left(K_{A}v_{B}-K_{B}v_{A}\right)}\right]\frac{bT_{P}}{v_{A}}-\left[\frac{K_{A}v_{B}-K_{B}v_{A}}{v_{A}-v_{B}}\right]\right\}}\equiv e_{A}^{*}, \end{array}$$

assuming that *T*_*N*_ is sufficiently large and *m* < (*K*_*A*_*v*_*B*_ − *K*_*B*_*v*_*A*_)/(*K*_*A*_ − *K*_*B*_). This indicates when litter decomposition rate of *P*_*A*_ is sufficiently low, the soil nutrient pool size of *P*_*A*_-cultivated soils is not maintained at higher levels, and consequently cannot prevent *P*_*B*_ invasion (which is assumed to be competitively superior under low soil nutrients). Similarly, *f*_*B*_(*N*${}_{B}^{*}$) has a root satisfying Equation (16), and thus *P*_*A*_ can invade, when *f*_*B*_($\hat{N}$) < 0. This leads to the following inequality:

(19) $$\begin{array}{r} e_{B}>\frac{mbT_{P}v_{B}\left[\left(K_{A}v_{B}-K_{B}v_{A}\right)-m\left(K_{A}-K_{B}\right)\right]}{v_{B}\left(K_{A}v_{B}-K_{B}v_{A}\right)\left\{T_{N}-\left[\left(v_{B}-m\right)-\frac{\left(v_{A}-v_{B}\right)K_{B}m}{\left(K_{A}v_{B}-K_{B}v_{A}\right)}\right]\frac{bT_{P}}{v_{B}}-\left[\frac{K_{A}v_{B}-K_{B}v_{A}}{v_{A}-v_{B}}\right]\right\}}\equiv e_{B}^{*}, \end{array}$$

suggesting that *P*_*A*_ can invade ***E***_*B*_ when litter decomposition rate of *P*_*B*_ is sufficiently fast (i.e., *e*_*B*_ > *e*${}_{B}^{*}$). When *e*_*B*_ > *e*${}_{B}^{*}$, the soil nutrient pool size of *P*_*B*_-cultivated soil is maintained at higher levels and provides *P*_*A*_ a growth advantage during invasion. When the two invasibility criteria are combined, the parameter space of *e*_*A*_ and *e*_*B*_ is categorized into four regions (Figure 2B). Coexistence (region C in Figure 2B) is possible when litter produced by *P*_*A*_ decomposes at a sufficiently slower rate, but litter of *P*_*B*_ decomposes sufficiently faster (i.e., *e*_*A*_ < *e*${}_{A}^{*}$, and *e*_*B*_ > *e*${}_{B}^{*}$). Dominance of *P*_*A*_ (region A in Figure 2B) occurs when *e*_*A*_ < *e*${}_{A}^{*}$, and dominance of *P*_*B*_ (region B in Figure 2B) occurs when *e*_*B*_ > *e*${}_{B}^{*}$. When litter produced by *P*_*A*_ decomposes at a sufficiently faster rate and *P*_*B*_ litter decomposes sufficiently slow (i.e., *e*_*A*_ > *e*${}_{A}^{*}$, and *e*_*B*_ < *e*${}_{B}^{*}$), the system has alternative stable states of either ***E***_*A*_ or ***E***_*B*_, depending on the initial plant species density (region A or B in Figure 2B).

### The effectiveness of invasion analysis and a revised PSF index

Based on invasibility analysis, we derived coexistence criteria for both microbial- and litter-mediated PSF models. Invasiveness of one plant species depends on how the resident-cultivated soil affects the growth of the invader compared to the resident, and the competition outcome is then assessed by the mutual invasion between plant species. Invasion is possible when the resident-cultivated soil microbial community has less positive effect on the resident species (e.g., α_*A*_ – α_*B*_ < 0 for microbial-mediated PSF), or when nutrient cycling has reduced benefit for the resident species (e.g., *e*_*A*_ < *e*${}_{A}^{*}$ for litter-mediated PSF). The rationale for these criteria is consistent with theoretical models, which indicate invasiveness is determined by the growth response of the invader in resident soil (Eppstein and Molofsky, 2007; Turnbull et al., 2010; Suding et al., 2013).

**Figure 3:**
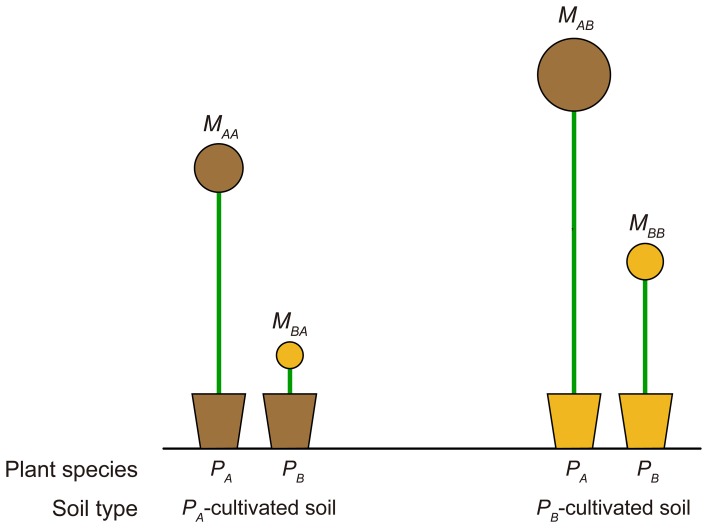
**Schematic diagram for a comparison between a traditional “plant-centered PSF index” and the revised “soil-centered PSF index,” which was an extension of invasibility analysis**. Plant-centered PSF index for plant species *A* (i.e., *P*_*A*_) is calculated as log(*M*_*AA*_/*M*_*AB*_), which compares growth response of *P*_*A*_ in *P*_*A*_- and *P*_*B*_-cultivated soils. The soil-centered PSF index for *P*_*A*_ is calculated as log(*M*_*AA*_/*M*_*BA*_), which compares the growth response of *P*_*A*_ and *P*_*B*_ in *P*_*A*_-cultivated soil. *M*_*ij*_ represents the growth response of plant species *i* in soil cultivated by plant species *j* (see main text).

PSF strength for a target plant species is commonly quantified in empirical studies by comparing the growth response of the target plant species in soils cultivated by different species (i.e., a “plant-centered PSF index”). For example, let *M*_*ij*_ represent the growth response of plant species *i* in plant species *j* soil; the plant-centered PSF index is commonly calculated as log(*M*_*ii*_/*M*_*ij*_). The invasibility analysis result provided here, which suggested that the competition outcome depended on how the resident-cultivated soil influenced the invader's growth compared with that of the resident, indicated the need for a revision of this metric. Based on the analytical results provided in the previous section, we argue the PSF quantified by comparing the competing plant species' growth responses in the target plant-cultivated soil were a better predictor for competition outcomes (i.e., a “soil-centered PSF index” for plant species *i*, calculated as log(*M*_*ii*_/*M*_*ji*_)). These soil-centered indices have been used in studies regarding a home-field advantage in litter decomposition (e.g., Ayres et al., 2009; Milcu and Manning, 2011), but to the best of our knowledge, not yet in empirical PSF studies. Figure 3 provides an illustrative example why the revised metric should be considered. Application of the commonly used PSF metric indicated that PSF for *P*_*A*_ was negative (i.e., log(*M*_*AA*_/*M*_*AB*_) < 0), while that for *P*_*B*_ was positive (i.e., log(*M*_*BB*_/*M*_*BA*_) > 0), predicting a *P*_*B*_ monoculture. However, in this example *P*_*A*_ was outcompeting *P*_*B*_ in soil environment cultivated by both *P*_*A*_ and *P*_*B*_. The actual competition outcome should be the competitive exclusion of *P*_*B*_ by *P*_*A*_, which can be accurately predicted by the soil-centered PSF index derived from the invasibility analysis (i.e., soil-centered PSF index for *P*_*A*_ was log(*M*_*AA*_/*M*_*BA*_) > 0, while that for *P*_*B*_ was log(*M*_*BB*_/*M*_*AB*_) < 0). In fact, out of all 24 possible plant growth response outcomes of a two-plant transplant experiment, the traditional plant-centered PSF index only correctly predicted half of the competitive outcomes, while the soil-centered PSF index correctly predicted all outcomes (see Figures S1–S4). However, we noted that when comparing growth response of different plants, biomass should first be normalized against their growth in uncultivated soils lacking plant growth (e.g., field-collected bare soil or autoclaved soils) to examine intrinsic biomass differences between different plant species. This reference soil choice can be critical, and sometimes challenging, however we believe studies can reveal a higher controlling power of PSF on community structure by quantifying PSF strength using a revised PSF index.

## Future directions

### Toward an integration of microbial- and litter-mediated PSF

The majority of PSF modeling papers to date has specifically addressed a single PSF mechanism or were phenomenological studies and did not assume a specific mechanism. The influence of PSF on plant and microbial communities can be better predicted by integrating microbial- and litter-mediated PSF in future research (de Deyn et al., 2004; Manning et al., 2008). Bever et al. (2010) reviewed the role of root-associated microbes in structuring plant resource partitioning, however plant nutrient uptake was not coupled with nutrient cycling in their framework. The role of saprophytic microbes in litter-mediated PSF is an interesting and important starting point to combine the two mechanisms, as microbial decomposers ultimately drive litter decomposition (Knops et al., 2002; Miki et al., 2010; Miki, 2012). The microbial decomposer community has been emphasized during discussions of differential litter decomposition rates at different sites (Ayres et al., 2009; Austin et al., 2014), and predictions of carbon storage in response to climate change (Allison et al., 2010; Wieder and Allison, 2013). However, the effects of decomposer community structure on plant competition outcomes are rarely explicitly discussed within the context of PSF. Recent theoretical studies revealed that community structure of microbial decomposers had critical consequences when predicting plant community structure and their responses to anthropogenic disturbance via litter-mediated PSF (Miki et al., 2010; Miki, 2012). In particular, Miki et al. (2010) demonstrated that functional diversity of microbial decomposers can weaken the positive litter-mediated PSF controlled by plant litter quality, thus facilitating plant coexistence.

Microbial decomposers are engaged in a broad range of interactions, which also provides linkages between litter- and microbial-mediated PSF. For example, in addition to providing nutrients via their active control on litter decomposition, microbial decomposers are directly involved in nutrient competition with plants (i.e., through immobilization). The competition strength depends on the plant's nutrient uptake strategies and litter quality (Daufresne and Loreau, 2001; Schimel and Bennett, 2004; Ushio et al., 2009). Miki et al. (2010) demonstrated these traits therefore influence the outcome of litter-mediated PSF, other than simply altering decomposition rates. Root-associated microbes, which were more commonly discussed in microbial-mediated PSF, might also indirectly influence litter-mediated PSF. For example, the competition between plants and microbial decomposers could be modified when mycorrhizal fungi have the ability to take up organic nutrients. Studies have reported nutrient uptake by ectomycorrhizal and ericoid mycorrhizal fungi, which resulted in decomposer carbon starvation and hence slower litter decomposition rates accompanied by increased soil carbon storage (Read and Perez-Moreno, 2003; Orwin et al., 2011; Averill et al., 2014). In addition to indirect modification of litter decomposition rates, root-associated microbes also altered plant litter input through their effects on plant productivity (Mitchell, 2003; van der Heijden et al., 2008; Orwin et al., 2011; Ke et al., 2015). In conclusion, the functional composition and community dynamics of microbes can interact with litter-mediated PSF, even when the microbes themselves are not directly engaged in litter decomposition. Our studies support an explicit consideration of the interdependency between the two PSF mechanisms, which can provide new predictions related to both plant and microbial community dynamics.

### Predicting species' PSF strength through a trait-based ecological approach

PSF acts as a structuring force in plant and microbial communities, therefore one important task for ecologists is to improve our prediction of species' PSF strength. The trait-based approach is a promising new approach, which has been widely applied to study plant community assembly via aboveground interactions (McGill et al., 2006; de Bello et al., 2010; Adler et al., 2013). Recent studies also applied trait-based approaches to elucidate microbial community assembly and the evolution of microbial life forms (Aguilar-Trigueros et al., 2014; Crowther et al., 2014). However, attempts to apply such approach remain scarce for PSF studies (van der Putten et al., 2013) (but see Baxendale et al., 2014). Ke et al. (2015) employed a trait-based approach to a theoretical PSF study, which assessed the sensitivity of PSF strength to various plant and microbial traits. Results revealed that the relative importance of litter decomposability compared to other plant demographic traits in determining PSF strength changes with the community composition of root-associated microbes. Baxendale et al. (2014) provided the first empirical data that associated PSF with plant foliar traits mentioned by the leaf economic spectrum (Wright et al., 2004). Results suggested that trait-based plant classification was most informative when plants are grown in mixtures (i.e., under interspecific competition), and plants responded positively to nutrient environments cultivated by plants with similar traits. One ultimate goal for trait-based studies is to predict shifts in ecological processes, such as PSF along abiotic gradients (McGill et al., 2006). The above studies suggested some traits might lose their impact along the abiotic gradient, as trait influence was also determined by biotic interactions with other plant and microbial species. We propose that additional studies linking plant and microbial traits to PSF strength or predicting species' PSF under different conditions are warranted.

### Combining PSF with general coexistence theory

Empirical and theoretical ecologists have long puzzled over the maintenance mechanisms of plant species coexistence. Chesson (2000) proposed that species coexistence was maintained by a combination of equalizing and stabilizing forces. This can be defined as equalizing forces that minimize differences in species' intrinsic growth rates, while stabilizing forces that limit species' per capita growth rates as species become common (Chesson, 2000; Adler et al., 2007). The establishment of a theoretical link between PSF-driven coexistence and classic coexistence theories is our final suggestion for future PSF research. Under Chesson's (2000) coexistence framework, PSF has been most commonly thought to act as a stabilizing mechanism by generating negative density-dependence (Bell et al., 2006; Yamazaki et al., 2008; Bagchi et al., 2010, 2014) or nutrient usage differentiation (Clark et al., 2005; Daufresne and Hedin, 2005; Bever et al., 2010). However, PSF can also facilitate plant coexistence through equalizing mechanisms. For example, if the competitive inferior species can form mutualistic associations with beneficial microbes (e.g., mycorrhizal fungi), which act in a density-independent manner, the presence of PSF can decrease fitness differences between competing species. Similar, equalizing examples in pathogens were also thoroughly reviewed (Mordecai, 2011, 2013a). If such equalizing forces of PSF are strong, plant coexistence can be maintained despite weak stabilizing forces by host specific pathogens or resource partitioning (Adler et al., 2007; Bever et al., 2010). The invasibility analysis provided in the present review is a viable starting point, as the invader's long-term low-density growth rate was one most important metric in Chesson's framework (Chesson, 2000). Future theoretical studies can continue separating the equalizing and stabilizing forces in current PSF models, and mechanistically link plant and microbial traits to the two coexistence mechanisms.

In conclusion, PSF research has attracted attention from many empirical ecologists and demonstrated notable success in using PSF to predict plant community patterns. Theoretical models, based on fundamental mechanistic-PSF models, have also substantially contributed to our knowledge. We believe the integration of multiple PSF mechanisms into the well-developed theoretical framework in ecology, such as stage-structure (e.g., Ke et al., 2015) and metacommunity (e.g., Loeuille and Leibold, 2014) models will produce new insights to understand plant and microbial community structure and dynamics.

### Conflict of interest statement

The authors declare that the research was conducted in the absence of any commercial or financial relationships that could be construed as a potential conflict of interest.
