# Supplementary material for: Incorporating the soil environment and microbial community into plant competition theory
Source: Front Microbiol. 2015 Oct 8;6:1066. doi: 10.3389/fmicb.2015.01066 (PMC4597134; doi:10.3389/fmicb.2015.01066)
Supplement: Supplementary file 1 [file Presentation1.PDF]

Incorporating the soil environment and microbial community into plant competition theory

Po-Ju Ke[1,2*], **Takeshi Miki**[2,3]

***Correspondence:** Po-Ju Ke: pojuke@stanford.edu

Supplementary Figure

The applicability of the revised soil-centered PSF index to a two-plant transplant experiment. All 24 possible scenarios of plant growth response are considered (Fig. S1-S4). The number marked on each plant represents the ranking of its growth response, with 1 representing the largest growth response. One can predicted the competition outcome based on the growth response of plants. The competition outcome can be classified into four types: monoculture of plant A (Fig. S1), monoculture of plant B (Fig. S2), alternative stable state of either plant A or plant B (Fig. S3), and coexistence of both plant A and B (Fig. S4). The plant-centered PSF index for species i, calculated as $\log(M_{ii}/M_{ij})$, can only successfully predict the community outcomes if half of the scenarios (see Fig. S1-S4 legend for detail). The revised soil-centered PSF index for species i, calculated as $\log(M_{ii}/M_{ji})$, can successfully predict the community outcome in all scenarios. M_{ij} represents the growth response of plant species i in soil cultivated by plant species j. See main text for further description.

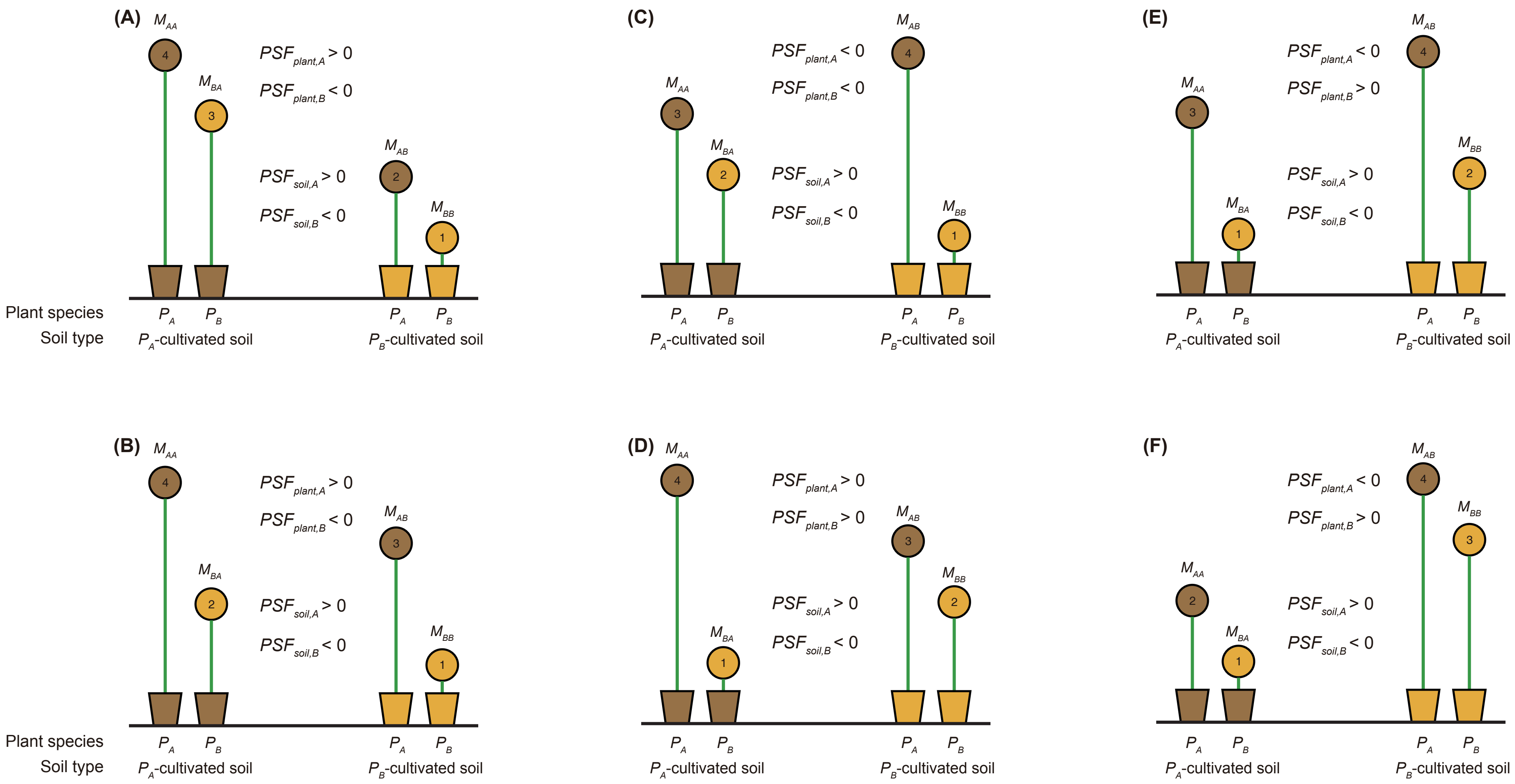

Fig. S1. The applicability of the revised soil-centered PSF index to a two-plant transplant experiment. All 6 possible plant growth scenarios that lead to the prediction of plant A monoculture are considered (panel A-F). The number marked on each plant represents the ranking of its growth response, with 1 representing the largest growth response. The expected feedback strength should be positive for plant A and negative for plant B. The plant-centered PSF index for species can only successfully predict the community outcomes in two scenarios (panel A and B). The revised soil-centered PSF index can successfully predict the community outcome in all scenarios (panel A-F). See main text for further description.

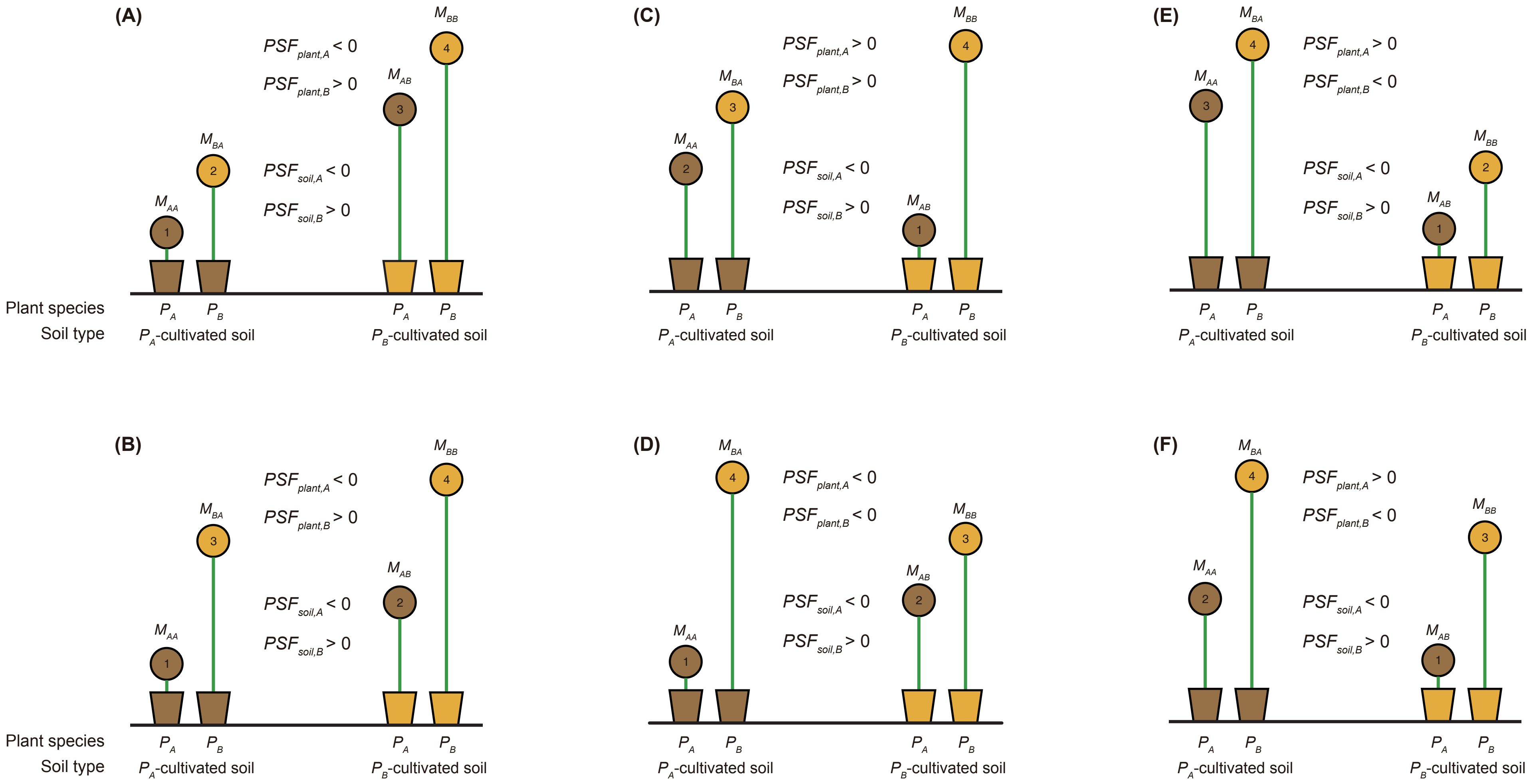

Fig. S2. The applicability of the revised soil-centered PSF index to a two-plant transplant experiment. All 6 possible plant growth scenarios that lead to the prediction of plant B monoculture are considered (panel A-F). The number marked on each plant represents the ranking of its growth response, with 1 representing the largest growth response. The expected feedback strength should be negative for plant A and positive for plant B. The plant-centered PSF index for species can only successfully predict the community outcomes in two scenarios (panel A and B). The revised soil-centered PSF index can successfully predict the community outcome in all scenarios (panel A-F). See main text for further description.

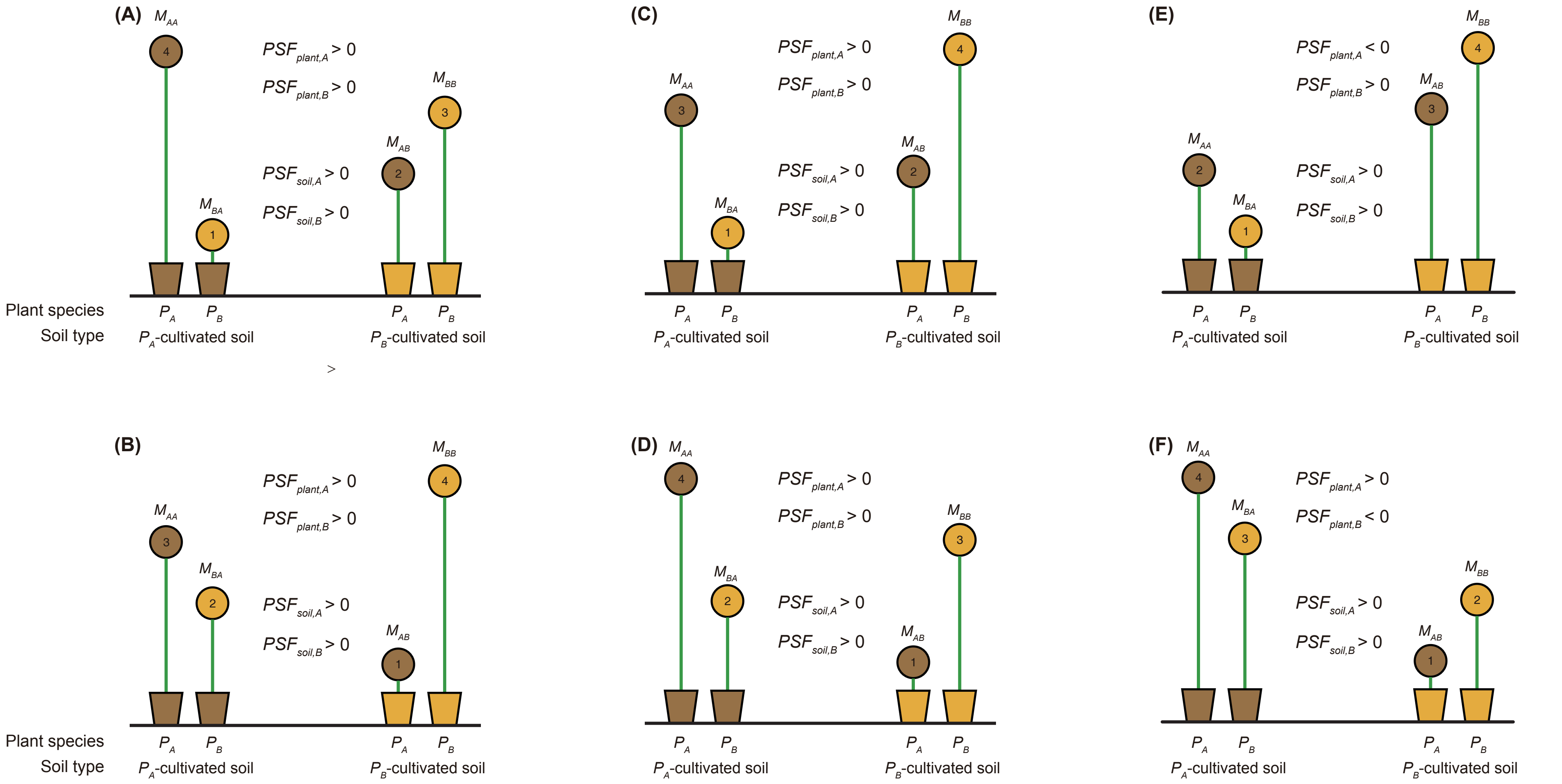

Fig. S3. The applicability of the revised soil-centered PSF index to a two-plant transplant experiment. All 6 possible plant growth scenarios that lead to the prediction of alternative stable states (i.e. monoculture of either plant A or plant B) are considered (panel A-F). The number marked on each plant represents the ranking of its growth response, with 1 representing the largest growth response. The expected feedback strength should be positive for plant A and positive for plant B. The plant-centered PSF index for species can only successfully predict the community outcomes in four scenarios (panel A-D). The revised soil-centered PSF index can successfully predict the community outcome in all scenarios (panel A-F). See main text for further description.

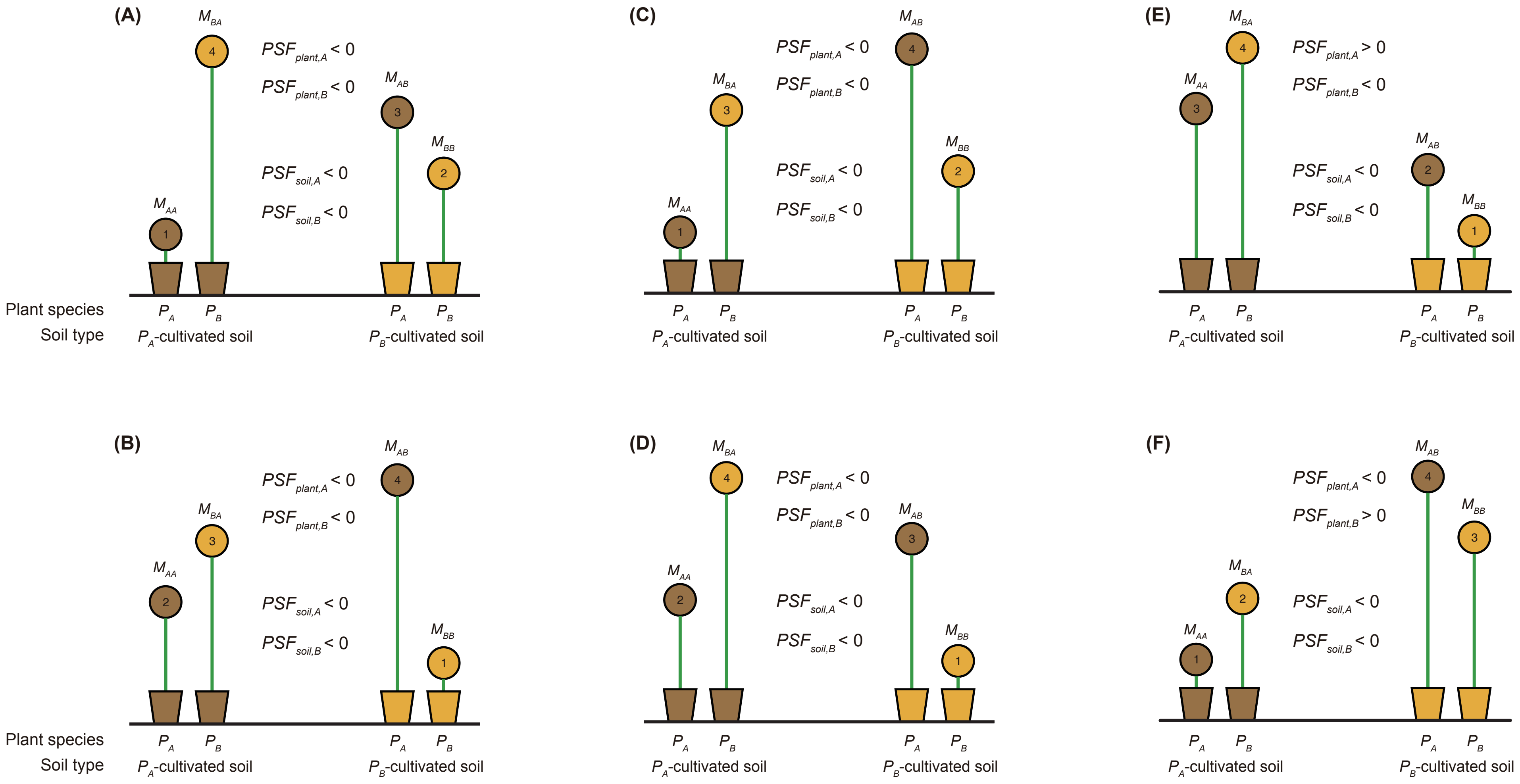

Fig. S4. The applicability of the revised soil-centered PSF index to a two-plant transplant experiment. All 6 possible plant growth scenarios that lead to the prediction of coexistence of both plant A and plant B are considered (panel A-F). The number marked on each plant represents the ranking of its growth response, with 1 representing the largest growth response. The expected feedback strength should be negative for plant A and negative for plant B. The plant-centered PSF index for species can only successfully predict the community outcomes in four scenarios (panel A-D). The revised soil-centered PSF index can successfully predict the community outcome in all scenarios (panel A-F). See main text for further description.
